# A valued voice: A qualitative analysis of parental decision‐making preferences in emergent paediatric surgery

**DOI:** 10.1111/hex.13686

**Published:** 2022-12-08

**Authors:** Erica M. Carlisle, Laura A. Shinkunas, Emily Ruba, Caleb J. Klipowicz, Maxwell T. Lieberman, Richard M. Hoffman, Heather S. Reisinger

**Affiliations:** ^1^ Department of Surgery, Division of Pediatric Surgery University of Iowa Hospitals and Clinics Iowa City Iowa USA; ^2^ College of Medicine, Program in Bioethics and Humanities University of Iowa Carver College of Medicine Iowa City Iowa USA; ^3^ College of Medicine University of Iowa Carver College of Medicine Iowa City Iowa USA; ^4^ Department of Anthropology University of Iowa Iowa City Iowa USA; ^5^ Department of Internal Medicine University of Iowa Hospitals and Clinics Iowa City Iowa USA; ^6^ Institute for Clinical and Translational Science University of Iowa Iowa City Iowa USA

**Keywords:** decision‐making, emergent, paediatric surgery

## Abstract

**Introduction:**

Shared decision‐making, with an emphasis on patient autonomy, is often advised in healthcare decision‐making. However, this may be difficult to implement in emergent settings. We have previously demonstrated that when considering emergent operations for their children, parents prefer surgeon guidance as opposed to shared decision‐making. Here, we interviewed parents of paediatric patients who had undergone emergent operations to better understand parental decision‐making preferences.

**Methods:**

Parents of paediatric patients who underwent surgery over the past 5 years at a University‐based, tertiary children's hospital for cancer, an emergent operation while in the neonatal intensive care unit (NICU) or extracorporeal membrane oxygenation (ECMO) were invited to complete a 60‐min semi‐structured interview. Interviews were digitally recorded and transcribed verbatim. Thematic content analysis was performed via deductive and inductive analysis. An iterative approach to thematic sampling/data analysis was used.

**Results:**

Thematic saturation was achieved after 12 interviews (4 cancer, 5 NICU and 3 ECMO). Five common themes were identified: (1) recommendations from surgeons are valuable; (2) ‘lifesaving mode’: parents felt there were no decisions to be made; (3) effective ways of obtaining information about treatment; (4) shared decision‐making as a ‘dialogue’ or ‘discussion’ and (5) parents as a ‘valued voice’ to advocate for their children.

**Conclusions:**

When engaging in decision‐making regarding emergent surgical procedures for their children, parents value a surgeon's recommendation. Parents felt that discussion or dialogue with surgeons defined shared decision‐making, and they believed that the opportunity to ask questions gave them a ‘valued voice’, even when they felt there were no decisions to be made.

**Patient or Public Contribution:**

For this study, we interviewed parents of paediatric patients who had undergone emergent operations to better understand parental decision‐making preferences. Parents thus provided all the data for the study.

## 1 INTRODUCTION

Thoughtful, effective engagement of patients in decision‐making has been shown to decrease decisional conflict (i.e., level of discomfort with the decision) and reduce overutilization of healthcare resources.^1^ Decision quality, the extent to which decisions are both informed and congruent with the patient's values, is also increased when patients are engaged in the decision‐making process.^1^ Given the elevated risks of morbidity and mortality as well as the potential emotional burden associated with discussions surrounding complex, high‐risk operations, meaningful engagement of patients in the decision‐making process may be especially important for patients considering such operations. Shared decision‐making (SDM), a process in which physicians and patients develop mutually agreed upon care plans that incorporate clinical evidence as well as patients' values and preferences, is generally considered to be a favourable approach to clinical counselling.^2^ However, relatively little data exists regarding the effectiveness of SDM for surgical counselling.^3^

Within the field of paediatric surgery, even fewer data are available regarding attitudes towards SDM or overall parental decision‐making preferences.^4^ Most available data focus on parental decision‐making preferences for elective paediatric surgical procedures.^4^ For example, Hong et al. evaluated the decision‐making process of parents considering elective otoplasty, a surgical procedure to correct prominent ears, for their children. The authors found that overall, parents experienced significant decisional conflict when considering this operation. However, those parents who considered themselves to have greater involvement in the decision‐making process reported less decisional conflict and regret than did parents who considered themselves to be less involved in the decision‐making process.^5^ Similar findings were echoed in a study of parents considering an elective tonsillectomy, adenoidectomy or tympanostomy tube insertion for their children. Parents who considered themselves to be more involved in the decision‐making process reported less decisional conflict when deciding whether their children should undergo surgery.^6^ While studies such as these suggest that parental involvement in the elective surgical decision‐making process is important, they offer limited insight into the specific components of the decision‐making process that promote parental involvement.

Parental decision‐making preferences for elective surgery may be different than those for emergent surgery. Parental preferences for emergent surgical decision‐making have not been extensively studied.^4^ In a survey of parents of paediatric surgery patients, we found that parental decision‐making preferences for paediatric surgery are context‐dependent.^7^ Specifically, we identified that parents prefer more guidance from surgeons when making decisions about operations they consider emergent as opposed to operations that they consider nonemergent.^7^ Marsh et al.^8^ had similar findings when they interviewed parents of children who sustained a life‐threatening, traumatic brain injury on their preference for who should make the decision to proceed with an operation to place an intracranial pressure monitor. While parents desired information and communication from physicians regarding the procedure, they overwhelmingly preferred that physicians decide whether to perform the procedure.^8^ These studies suggest that parents increasingly prioritize guidance from their child's surgeon when making emergent decisions; however, further investigation of parental decision‐making preferences is needed to fully understand this issue.

Enhancing our understanding of parental decision‐making preferences in emergent paediatric surgical settings will help surgeons communicate with parents in a way that appropriately promotes parental involvement, facilitates trust and minimizes decisional conflict and regret. We conducted semistructured interviews with parents of paediatric patients who had undergone emergent operations to identify common themes regarding their decision‐making preferences.

## 2 METHODS

### 2.1 Study design and recruitment

We conducted semi‐structured interviews to broadly explore parents' experiences and preferences towards decision‐making in three emergent paediatric surgical settings at a university‐based children's hospital.^9^, ^10^ The study was approved by the University of Iowa Institutional Review Board (#201905811) and is reported according to the Consolidated Criteria for Reporting Qualitative Research (COREQ) checklist (Appendix 1).^11^ All methods were carried out in accordance with relevant guidelines and regulations. As approved by the University of Iowa Institutional Review Board, informed consent was obtained verbally at the commencement of each interview. All participants were over the age of 18.

We used a purposive sampling technique to identify parents of paediatric patients (<18 years old) who had undergone surgery by a paediatric surgeon for major resection of a solid tumour, an urgent operation while in the neonatal intensive care unit (NICU) or extracorporeal membrane oxygenation (ECMO) between 2015 and 2020. ECMO was included, as this is a procedure performed only when patients are in immediately life‐threatening clinical circumstances. Solid tumour resection operations and operations on neonates in the NICU were selected based upon a prior survey conducted by our research team where parents of paediatric surgical patients were provided six hypothetical paediatric surgical scenarios and asked to identify the operations they considered emergent.^7^ Parents identified operations for cancer and operations on babies to be emergent operations.

Eligible parent participants were sent an initial email or letter, which was followed with a phone call if there was no response after 2 weeks. Parent participants were also recruited through our hospital's Facebook page and Twitter feed. To maintain our purposive sampling technique, interested parents were asked to complete a brief online survey to ensure that their child met the eligibility criteria. Participants received a $20 Amazon gift card after the completion of the interview.

### 2.2 Interview procedures

Semistructured interviews were conducted by phone between June 2019 and April 2020. All interviews were conducted by a research specialist (L. A. S.) or a medical anthropologist (C. J. K. or M. T. L.) trained in the process of semistructured interviews. We designed a semistructured interview guide of 20 open‐ended questions based on the literature review and our prior survey data (Appendix 2). During the interview, parents were asked to create an illness narrative about their child's surgery. The questions sought to explore ‘personal experience narratives’ by asking parents about the events leading up to their child's surgery, the process of making surgical decisions, and the consequences of the decisions that were made.^12^ The illness narrative specifically emphasized the amount of guidance and interaction parents desired and experienced from the surgeon, the amount of involvement in the decision‐making process parents preferred and experienced, how much procedural detail or information regarding procedural risks was desired and received, preferred approaches for receiving information and feelings regarding the outcome of the surgery and the surgeon. At the end of the interview, parents were asked what the term ‘shared decision‐making’ meant to them. There were no question prompts. The main questions in the interview guide were intended to direct the conversation. However, the interview was not limited to the questions in the guide but rather shaped by each parent's responses to the main questions. Throughout the interview process, questions were refined based on the consensus of the research group as well as concurrent iterative analysis.

### 2.3 Data analysis

Interviews were digitally recorded on a secure device. Audiotapes were transcribed verbatim by a member of the research team and de‐identified. Thematic content analysis was performed using deductive codes from the interview guide and the literature and inductive codes that were identified in the data.^10^ An iterative approach to thematic sampling and data analysis was used. Three members of our research team (L. A. S., M. T. L. and E. M. C.) met weekly to discuss the overall progress of the project. This team coded all transcripts using a deductive and inductive approach, which involved constant comparative analysis and multiple iterations of coding as the research team oscillated between gathering and analysing the data. A codebook was developed and repeatedly refined based on group discussion of the data. Each research team member independently coded the transcripts before all discussions or refinement of the codebook. Constant comparative analysis continued until data saturation, the stage where ‘there are mounting instances of the same codes, but no new ones’ was reached.^13^ Coding was reviewed and saturation was assessed after completion of every two to three interviews. In total, we performed 12 interviews (4 cancer, 5 NICU and 3 ECMO). Transcripts were uploaded into MAXQDA 2020 (Verbi Software, 2019) for data management and analysis of themes across interviews.

## 3 RESULTS

### 3.1 Participants

Twelve parents of 12 paediatric surgical patients were interviewed. All participants were mothers of children who had undergone ECMO, an emergent operation while in the NICU or major resection of a solid tumour. Patient demographics are presented in Table 1. Parental race, ethnicity and socioeconomic status were not collected. Interview length ranged from 25 to 65 min.

**Table 1:** Patient demographics

Participant ID	Surgical category	Patient sex	Patient status	Patient's diagnosis	Patient age at first surgery
P1	Cancer	F	Alive	Wilms tumour	2 years old
P2	Cancer	F	Alive	Wilms tumour	3 years old
P3	Cancer	F	Alive	Wilms tumour	5 years old
P4	Cancer	F	Alive	Neuroblastoma	2 years old
P5	ECMO	M	Alive	Persistent pulmonary hypertension	1 day old
P6	ECMO	F	Alive	Congenital diaphragmatic hernia	9 days old
P7	ECMO	M	Alive	Sepsis	13 years old
P8	NICU	F	Alive	Bowel perforation	19 days old
P9	NICU	M	Deceased	Bowel perforation	10 days old
P10	NICU	M	Alive	Necrotizing enterocolitis	12 days old
P11	NICU	M	Alive	Persistent pulmonary hypertension	1 month old
P12	NICU	F	Alive	Gastric perforation	3 days old

Abbreviations: ECMO, extracorporeal membrane oxygenation; F, female; M, male; NICU, neonatal intensive care unit.

### 3.2 Common themes

Five common themes were identified: (1) recommendations from surgeons are valuable; (2) ‘lifesaving mode’: parents felt there were no decisions to be made; (3) effective ways of obtaining information about treatment; (4) shared decision‐making as a ‘dialogue’ or ‘discussion’ and (5) parents as a ‘valued voice’ to advocate for their children. Themes did not vary based on the type of surgery the patient underwent or the age of the patient.

#### 3.2.1 Theme 1: Recommendations from surgeons are valuable

The majority of parents (8 out of 12) specifically noted that receiving a recommendation about surgical intervention from a surgeon was valuable. Surgeons were described as ‘the experts [who] knew what needed to be done’ (P11). As one parent articulated, ‘I mean alright if we do A, B and C, then her survival rate is XYZ. For me, I found that helpful’ (P3). Parents explained that recommendations provided a sense of confidence in the care plan and made them feel involved in the process of caring for their children. As one parent pointed out, ‘ultimately the surgeon needs to share what they're recommending and how they want to proceed’ (P4). Another parent explained that the recommendation ‘kind of gave me something to look up online to try to figure out [things]’ (P12). Not all surgeon recommendations were perceived as helpful. One parent expressed that the recommendation provided was not helpful due predominantly to the blunt manner in which it was delivered by the surgeon, suggesting that a surgeon's approach to communication may influence parent's perceptions of recommendations as well.

#### 3.2.2 Theme 2: ‘Lifesaving Mode’: Parents felt there were no decisions to be made

All parents independently expressed that they felt as if there were no surgical decisions to be made. No specific question was asked by interviewers to assess this sentiment, rather this feeling was organically identified by parents during every interview. As one cancer parent stated, ‘When she first was diagnosed, they made all the decisions. They told me what was gonna happen’ (P1). An ECMO parent acknowledged, ‘No, there was no decision. We were all just in just lifesaving mode’ (P5). Parents tended to express the belief that consenting to further care was seen not as a decision but as the best option. As one parent articulated, ‘I don't feel like we made that many decisions. It was just you know being guided through by the doctors and nurses. Like doing the next best thing. This is the next right thing to choose’ (P7). Death of the patient or transition to comfort measures were not seen as reasonable choices by any of the parents interviewed. For instance, one NICU parent indicated, ‘That was one of those things where there were really no options. You either had to do surgery or death’ (P10). Another parent shared this sentiment by stating, ‘It was this or a funeral. There wasn't any other option’ (P5).

#### 3.2.3 Theme 3: Effective ways of obtaining information about treatment

All parents acknowledged the importance of direct communication with surgeons and other providers.

I know that it's a very involved process having a sick child. But I really appreciated the fact that the surgeon sat down with us, and discussed everything they were going to do. They told us about the surgery in detail. They told us exactly what medications he was going to be on. And they were completely honest with us as far as what his chances of survival were. (P9)

Three parents emphasized the importance of a slow conversational pace with significant repetition. As one parent expressed, ‘It's easy to trust [the surgeon] when you feel like you're not being rushed out the door or rushed with any questions or concerns that you have and you know that they want to help you’ (P12). Another parent verbalized, ‘it was reassuring to hear a story three times so that we could actually gather the facts’ (P7). Most parents also highlighted their appreciation of the surgeons' efforts to use drawings and other nonverbal, physical means of communication to clarify explanations. Specifically, four parents emphasized the favorability of a protocol or guidebook, four parents highlighted the use of images and diagrams and two parents emphasized the use of brochures or other printed materials. In reference to the helpfulness of images and diagrams, one parent stated, ‘She drew pictures, and it made so much sense to me’ (P7). Thinking about the helpfulness of a protocol or guidebook, one parent said:

Back in the beginning when she first was diagnosed I was really glad that they gave me this big old notebook with all this information and it had that you know that protocol in there telling me what was gonna happen and when she was gonna have chemo and how many weeks apart they were and everything. And I think that was comforting … because you never know what's gonna happen but at least you have that timeline and you can kind of try to figure out your life with the timeline of chemo and surgery and whatever. (P1)

Two parents specifically emphasized the importance of being provided with as much information as possible ‘…regarding risks, outcomes, benefits, possible complications, and then what the plan would be if something were to happen. Just so that if something does happen, you don't feel like you're in the dark’ (P8). Only two parents highlighted the use of statistics or descriptions of the surgical procedure in their assessment of useful information. Several parents expressed that poor surgeon demeanour (*n* = 1), repeated questions from multiple teams (*n* = 1) and acronyms (*n* = 1) were unhelpful forms of communication.

To supplement direct communication with surgeons, eight of the 12 parents discussed searching for information online. According to one parent, ‘everybody says don't go to Google or don't, you know, live on the internet. But sometimes that's the only recourse you have when it comes to not having the medical staff around you’ (P5). While one parent expressed that parents are ‘not supposed’ to do online searches, another parent was comforted by finding online evidence of international research on her child's diagnosis and another parent suggested, ‘…it made what the doctors said more understandable’ (P3).Three parents used social media to connect with other parents. Additionally, several parents (*n* = 4) suggested that virtual communication opportunities may enhance communication with the healthcare team. For instance, one parent noted that,

If there was a way that, instead of having to call in and see how rounds went when we couldn't be there, if there was a way that we could have a monitoring system, almost, in their rooms. And we could check in … it would be nice if it was something that we could log in knowing that rounds were gonna' be done between like 9 and 11 or whatever. And then they could like teleconference rounds. (P5)

#### 3.2.4 Theme 4: Shared decision‐making as a ‘dialogue’ or ‘discussion’

All parents were asked what the phrase ‘shared decision making’ meant to them. Overall, shared decision‐making was seen as a ‘dialogue’ or ‘open discussion between the surgeon and the family’ (P2). Nine parents highlighted the importance of participating in the decision‐making process. One parent defined shared decision‐making as ‘the doctor and the patient or the guardian you know agreeing upon what you're going to do. Not just the doctor coming in and saying this is what we're doing. Making the guardian or the parent or the patient feel involved in the decision‐making process’ (P4).

Five parents emphasized the importance of the surgeon's recommendation in the discussion.

If someone told me shared decision making, I would think that they were asking me to help make the decision on my child's treatment. I mean they're the experts. I mean so shared decision making, if you're saying so here's the recommendation and this is why I'm sharing with you my opinion why this is the best thing for your child, that's great. And I do want to know that. But at the end of the day, whatever they recommend, I'm gonna do it. (P3)

Three parents mentioned the importance of explaining options. For instance, one parent defined shared decision‐making as ‘…explaining the options if there are options, I guess. I think I would always prefer a recommendation and then want to know the reasons behind that recommendation’ (P2). Three parents said that the importance of shared decision‐making might depend on the person or the situation.

You could say every parent needs to have input, but some parents don't have anything to input because they don't understand what's going on. So, I don't know, it's not, it just varies on the situation with the family. It's not like a cut and dry thing. It's kind of complicated. I guess it all depends on the person. (P1)

Only one parent mentioned the importance of parent responsibility for making the final decision.

#### 3.2.5 Theme 5: Parents as a ‘valued voice’ to advocate for their children

All 12 parents relied on surgeons for information. Eight parents used internet searches and social media to supplement their knowledge. All parents expressed that even when they felt there was no specific decision to be made, the opportunity to use the information they had gathered to be involved in their child's care and included in discussions regarding their child was important. This engagement gave them a ‘valued voice’, which was described by one parent as:

I felt like a valued voice in her care plan…. [Interviewer asks what makes parent feel valued]…. Just that they actually listen to us. Like if we're asked a question about how she's doing or different things that were going on that they actually wanted to hear the answer instead of just asking to ask. And that they actually kind of took it to heart when making decisions. (P12)

All parents also identified the need to advocate for their children; however, the approach parents chose varied based upon self‐described personality characteristics. Three parents identified advocacy as speaking up when they perceived their child was being cared for incorrectly (including instances of wrong medication, wrong time to give medication or delayed stitch removal).

…like a couple times medications were given at the wrong times or given twice. Like if one nurse was busy, another nurse would come in and I would say, I think that was just given. You know and there were a couple times like that that I'm kind of glad that I got overly invested in it. (P7)

Four parents emphasized the importance of extending this advocacy to other patients or families. One parent shared that their family has ‘…stayed super involved with the NICU. We volunteer. We fundraise for them. We mentor some other parents now’ (P11). Another parent revealed, ‘I feel like we speak out more about stuff that we don't agree with like anti‐vaxxers because [she] was exposed to whooping cough from an unvaccinated child while she was on chemo’ (P3).

All parents emphasized the importance of gaining the knowledge that they needed to effectively advocate for their children. As one parent expressed, ‘Once we figured out what rounds were, about a week later, we were there every day taking notes, and I would say we were very interested in learning the science and medicine behind what was going on in case we had to make decisions’ (P11). Another parent conveyed, ‘…we wanted to go for the best option for her and we did a lot of talking and going back and forth with a couple different surgeons on the team to see, to get their different points of view’ (P12).

## 4 DISCUSSION

Our qualitative analysis of parental decision‐making preferences in emergent paediatric surgical settings has identified several key themes that can help paediatric surgeons promote parental involvement in ways that may facilitate trust and minimize decisional conflict and regret. Specifically, our work offers unique insight into parental perspectives on the relative value of autonomy, the perceived meaning of shared decision‐making and the ways in which parents prefer to communicate with surgeons. Interpretation of these themes guides specific suggestions for how surgeons may assure meaningful parental engagement in surgical decision‐making.

Much has been written about the prioritization of patient autonomy in healthcare decision‐making.^2^, ^14^ Some ethicists argue that physician efforts to guide patients towards particular clinical decisions violate respect for patient autonomy.^15^ Physicians have expressed concern that this seemingly rigid emphasis on patient autonomy creates encounters in which clinicians are expected to outline a spectrum of options that the patient may choose among as opposed to providing a recommendation.^16^ Our findings suggest that parents of paediatric surgical patients value recommendations from the surgeon when considering emergent surgery for their children. The parents we interviewed describe that the recommendations make them feel more confident and involved in caring for their children. They emphasize that the recommendation provides a concrete scaffolding to guide the further pursuit of the knowledge they need to participate in the care of their children in an informed way—without the burden of decision‐making. This finding supports an emerging body of literature that suggests that patients may prefer more guidance from physicians during decision‐making as opposed to a greater emphasis on autonomy.^8^, ^17^, ^18^

Over the past several decades, SDM has been highlighted as a process that can prioritize patient autonomy by encouraging patients and physicians to work together to develop care plans that account for patient goals and values while inviting patients to participate in decision‐making at a level they deem most appropriate.^2^ Studies using the Degner control preference scale, which evaluates a patient's desired level of involvement along a continuum of autonomous patient decision‐making to provider‐based decision‐making, suggest that most respondents favour some degree of collaboration with providers as opposed to a highly active or highly passive role in decision‐making.^19^, ^20^, ^21^ While this concept of shared decision‐making seems relatively straightforward to apply to clinical encounters, few studies have investigated what it means to patients to truly share in the surgical decision‐making process.^22^ Clarifying our understanding of how parents interpret the goals of SDM can help refine our approach to surgical counselling. The parents we interviewed emphasize that it is engagement in open discussion with the surgeon, as opposed to final decision‐making autonomy, that defines shared decision‐making. The parents in our study felt that they had engaged in SDM even though they unanimously felt that there were no decisions to be made. This apparent disconnect reinforces the notion that when considering emergent operations for their children, engagement and inclusion in the decision‐making process are what parents desire—not final decision‐making autonomy. Shared decision‐making frameworks that overly prioritize parent autonomy thus seem inappropriate in the context of emergent paediatric surgery. The emphasis on involvement in the decision‐making process has been highlighted in elective paediatric surgery decision‐making^5^, ^6^; however, the finding that this decision‐making preference exists for parents considering emergent operations for their children has not been widely described.

Identification of the specific behaviours that help parents feel most involved in the decision‐making process is a critical first step in assuring that surgeons promote the inclusion of these behaviours. The parents we interviewed expressed that the opportunity to ask questions about their child's care gave them a ‘valued voice’ even when they felt there was no specific decision to be made. This emphasizes the importance of providing ample opportunity for parents to ask questions throughout the decision‐making process. Work by Pecanac et al.^23^ suggests that patients may benefit from targeted guidance regarding how to assure pertinent, meaningful questions are asked during the surgical consultation. In their analysis of the types of questions patients asked before high‐risk surgical procedures, this group found that patients focused upon logistical or technical questions such as whether the incision would be closed with sutures or staples, how to wash their hair postoperatively and vehicle parking options during their hospital stay.^23^ While it is certainly important to consider all patient concerns, one may argue that time spent in consultation with the surgeon is best utilized to facilitate discussions of more critical issues such as complications of the procedure, risk of disease recurrence or risk of morbidity or mortality. Future work to develop a decision‐support tool that prepares parents to effectively ask questions, and thus exercises their ‘valued voice’, during surgical consultation may improve the decision‐making process for parents of children undergoing emergent operations.

A Question Prompt List (QPL) may be a well‐suited decision‐support tool for parents. QPLs are structured lists of questions that patients can use to identify meaningful questions to ask during discussions with clinicians.^24^ QPL use has been shown to improve communication across many healthcare disciplines, but QPLs have not been evaluated in paediatric surgery.^25^, ^26^, ^27^ Further work to identify the specific details parents and surgeons deem most critical to include during the decision‐making process will help guide the development of a QPL that parents can use when discussing emergent surgery for their children. The QPL may need to be modified to address specific operations and pathologies; however, many of the fundamental questions (What are the risks of surgery? Are there alternatives to this surgery? etc.) would likely be relevant regardless of the operation being performed. Consideration of the distinction that the parents we interviewed made between seeking information to promote informed involvement in the care of their children as opposed to seeking information to promote decision‐making preparedness will also help guide the intentional construction of a QPL. Analysis of surgeon preferences on the decision‐making process for emergent paediatric surgery will further aid in the meaningful construction of such a decision‐support tool and facilitate its integration into surgical practice.

Our work also demonstrates that while parents emphasized the importance of direct conversation with their child's surgeon to learn about the treatment, they also valued other information sources. Specifically, the parents we interviewed highlighted that reviewing written and graphical information (drawings, brochures, figures, etc.) helped promote their involvement in the decision‐making process. Additionally, all parents in our study relied on online resources to supplement their understanding of their child's illness. The parents we interviewed expressed mixed emotions about whether surgeons approved of their online exploration. This suggests that an opportunity exists for surgeons to guide online searches by providing reliable websites or preferred social media groups to parents. Such guidance may both quell parents' concerns that they are ‘not supposed to’ seek information online as well as help direct them to the most helpful and accurate online resources.

The parents we interviewed also suggested that increased opportunity for virtual communication adjuncts would facilitate their engagement in the decision‐making process. Specifically, parents suggested that having a virtual presence during surgical rounds (when they were not able to be physically present) would promote enhanced communication with surgeons. The COVID‐19 pandemic has brought with it increased focus on opportunities for telemedicine and virtual communication with patients and families.^28^ Further exploration of how virtual communication platforms can be integrated into surgical counselling may yield insight into how to most effectively integrate these platforms into the care of paediatric surgical patients.

Parents in our study also emphasized the importance of advocacy—both for their own children as well as for families of other children with similar surgical issues. Parents' advocacy for their children was often subtle, such as being present for rounds and asking questions. However, some parents described engagement in more direct advocacy to prevent incorrect doses of medication from being given to their children or medicines from being given at incorrect times. Multiple parents highlighted the importance of advocating for other families by means of social media or hospital‐sponsored programmes. Given the importance that parents place on these opportunities, surgeons should consider more directly providing parents with opportunities to advocate for their children as well as for families with children with similar surgical problems.

Our study has several limitations. Parent perspectives were solicited from a single, Midwestern, academic centre thereby limiting the generalizability of our results. Additionally, while the opportunity to interview was offered to both parents, only mothers volunteered to be interviewed. The inclusion of fathers or other guardians would increase the depth and generalizability of our analysis. Our work is also limited in that we did not solicit parent race, ethnicity, familial characteristics or socioeconomic status. This study was not designed to investigate thematic differences based on these variables, but subsequent studies should address these important factors. The voluntary nature of parental participation in our study may also have skewed results, in that those parents with less favourable attitudes towards the healthcare team may have been less likely to participate. Parents who chose to allocate their time to this study may also not be representative of all parents whose children are cared for in our institution and given that the researchers were from the same institution at which the children received surgical care, parents may have felt social pressure to respond positively during the interviews. Additionally, 11 of 12 parents in our study had living children at the time of the interview. The positive outcome for these children may have prompted parents to speak more highly of clinicians regardless of the quality of communication. Further, although grounded in prior work that evaluated parental interpretations of the urgency of given paediatric surgical operations,^7^ our classification of the selected clinical settings (ECMO, NICU and cancer) as emergent is relatively subjective. One must also consider that identification of Theme 2 (‘Lifesaving mode’: parents felt there were no decisions to be made) may have occurred due to incomplete informed consent discussions in which clinically reasonable options to transition to palliative care or forego surgical intervention were not offered. Given the diversity of the clinical encounters as well as the fact that patients of all paediatric surgeons at the institution were included in the analysis, it seems unlikely that these options would have been omitted during every informed consent discussion. It seems more likely that parents may have considered options resulting in the likely death of their children to be intolerable even if the options may have been clinically and ethically reasonable (i.e., choosing not to undergo ECMO cannulation).

Parental recall bias may also have impacted our data in that we conducted interviews with parents at variable times following their child's surgery. Standardizing the length of time between surgery and the interview may limit this bias. However, this approach may impair our ability to recruit a sufficient number of parents for interviews. Our patient cohort also had significant clinical heterogeneity. A more specific focus on patients with a single diagnosis who underwent a particular procedure may have yielded more precise data. Additionally, in our attempt to focus specifically on the interaction between parents and surgeons, we failed to explore the impact of other care providers in the decision‐making process. Although beyond the scope of the current study, future work should address how parents' interactions with oncologists, neonatologists and other care providers impact surgical decision‐making. Finally, we did strive to ensure internal validity through the inclusion of three different interviewers with varying nonclinical backgrounds, interviewing multiple parents, inductive reasoning during analysis, independent coding of each interview transcript by three research team members and use of verbatim quotes to justify identified themes; we did not discuss the identified themes with participants or seek participant feedback on the accuracy of our themes. The inclusion of such participant checking should be considered in subsequent studies.

## 5 CONCLUSIONS

Our qualitative analysis of parental decision‐making preferences during emergent surgical decision‐making demonstrates that parents value guidance from surgeons by way of a recommendation. The parents we interviewed believe that inclusion in their children's care, as opposed to decision‐making autonomy, is what defines shared decision‐making. They feel included when given the opportunity to ask questions and use the information that they gather to serve as a ‘valued voice’ to advocate for their children as well as for families of children with similar surgical conditions. The parents in our study appreciate physical supplements to verbal communication, and they value the opportunity for telemedicine or virtual engagement when direct, in‐person communication is not possible. Integration of these findings into surgical counselling will allow paediatric surgeons to modify their counselling for emergent procedures to optimize parental preferences. Such changes will promote parental involvement and are likely to facilitate trust and minimize decisional conflict and regret.

## AUTHOR CONTRIBUTIONS

*Conception and design of the study*: Erica M. Carlisle, Laura A. Shinkunas, Heather S. Reisinger. *Acquisition of data*: Erica M. Carlisle, Laura A. Shinkunas, Emily Ruba, Caleb J. Klipowicz, Maxwell T. Lieberman. *Analysis and interpretation of data*: Erica M. Carlisle, Laura A. Shinkunas, Emily Ruba, Maxwell T. Lieberman, Richard M. Hoffman, Heather S. Reisinger. *Drafting and revision of the article*: Erica M. Carlisle, Laura A. Shinkunas, Emily Ruba, Caleb J. Klipowicz, Maxwell T. Lieberman, Richard M. Hoffman, Heather S. Reisinger. *Final approval of the version to be submitted*: Erica M. Carlisle, Laura A. Shinkunas, Emily Ruba, Caleb J. Klipowicz, Maxwell T. Lieberman, Richard M. Hoffman, Heather S. Reisinger. All authors read and approved the final manuscript.

## CONFLICT OF INTEREST

The authors declare no conflict of interest.

## ETHICS STATEMENT

The study was approved by the University of Iowa Institutional Review Board (#201905811).

## Data Availability

Data that support the findings of this study are available from the corresponding author upon reasonable request.

## APPENDIX 1 CONSOLIDATED CRITERIA FOR REPORTING QUALITATIVE RESEARCH (COREQ) CHECKLIST

**Table:**

No.	Item	Description
Domain 1: Research team and reflexivity		
*Personal characteristics*		
1.	Interviewers(s)	Laura A. Shinkunas (L. A. S.), Caleb J. Klipowicz (C. J. K.), Maxwell T. Lieberman (M. T. L.)
2.	Credentials	C. J. K. (PhD candidate in anthropology); M. T. L. (PhD candidate in anthropology); L. A. S. (MS)
3.	Occupation	C. J. K. and M. T. L.: graduate research assistants; L. A. S: research specialist
4.	Gender	C. J. K. and M. T. L.: male; L. A. S.: female
5.	Experience and training	C. J. K.: Educational background in medical anthropology. Practical experience in qualitative research and teaching; M. T. L.: Educational background in cultural anthropology. Practical experience in qualitative research and teaching. L. A. S.: Educational background in psychology and bioethics. Practical experience in qualitative research.
*Relationship with participants*		
6.	Relationship established	No
7.	Participant knowledge of the interviewer	Participants knew that C. J. K. and M. T. L. were working as graduate research assistants with Erica M. Carlisle, a paediatric surgeon in the Department of Surgery.
8.	Facilitator characteristics	Participants knew that C. J. K. and M. T. L. had social science backgrounds.
Domain 2: Study design		
*Theoretical framework*		
9.	Methodological orientation and theory	Thematic content analysis (Bernard, 2016)
*Participant selection*		
10.	Sampling	Purposive sampling
11.	Method of approach	Email
12.	Sample size	12 participants
13.	Nonparticipation	Not applicable
*Setting*		
14.	Setting of data collection	Interviews were conducted by phone
15.	Presence of nonparticipants	NA
16.	Description of sample	Not collected
*Data collection*		
17.	Interview guide	Provided as supplemental material
18.	Repeat interviews	No
19.	Audio/visual recording	Audio recording
20.	Field notes	No
21.	Duration	Mean = 28.7 (range:25–65 min)
22.	Data saturation	Yes
23.	Transcripts returned	No
Domain 3: Analysis and findings		
*Data analysis*		
24.	Number of data coders	Three
25.	Description of the coding tree	Provided as supplemental material
26.	Derivation of the themes	Inductive and deductive (themes were derived from both previous literature and the interview data)
27.	Software	MAXQDA 2022 (Verbi Software, 2021)
28.	Participant checking	No
*Reporting*		
29.	Quotations presented	Yes
30.	Data and findings consistent	Yes
31.	Clarity of major themes	Yes
32.	Clarity of minor themes	Not applicable

## APPENDIX 2 INTERVIEW GUIDE. SEMISTRUCTURED INTERVIEW GUIDE

Interviewer:

Date:

Participant Code:

**Illness Narrative Overview**

Could you describe for me the events that led up to your child's surgery? (Diagnosis, series of events, age of the child, etc.)

Could you tell me about your child's operation? (e.g., What did the doctors do? What did you do during the procedure?)

What happened after the surgery?

How has your child been since then?

**Decision‐Making Process**

What did the doctor(s) tell you about the condition/surgery when you met with them? (e.g., did they offer you information about the surgery or condition, etc.?)

What were some of the decisions you had to make about your child's surgery?

What did you end up deciding to do? How did this make you feel?

How did you reach this decision? Who did you talk to help you make these decisions?

Do you think this process of reaching a decision would have been different if the treatment was for you and not your child?

**Surgeon–Parent Interactions**

How would you describe your interactions with the surgeon during the decision‐making process?

Did the surgeon make a recommendation to you about surgery?

If Yes—Was it important/helpful to you for your surgeon to offer you a recommendation?

How do you feel about the guidance the surgeons/doctors offered you? (e.g., Do you have strong feelings about it? Did they meet your expectations?)

How might your feelings be different about your surgeon's guidance if your child's surgery was less of an emergency?

**Reflection and Recommendations**

Overall, how do you feel about this experience? (e.g., satisfied? Regret?)

How do you feel about the outcome of the surgery?

Looking back, do you wish anything had been done differently?

After this experience, how would you recommend surgeons work with parents that are in similar situations to the one you faced?

Is there anything that you would change or improve about the process of making decisions about surgery for your child?

**Other/Wrap‐up**

Finally, in medicine, there is currently a push for doctors and surgeons to share in the decision‐making process with their patients. Could you tell me, what ‘shared decision making’ mean to you?

Is there anything else you think is important that I did not ask you about that you would like to share?

Do you have any questions for me?
